# Principles of cardiovascular magnetic resonance feature tracking and echocardiographic speckle tracking for informed clinical use

**DOI:** 10.1186/s12968-016-0269-7

**Published:** 2016-08-26

**Authors:** Gianni Pedrizzetti, Piet Claus, Philip J. Kilner, Eike Nagel

**Affiliations:** 1 Department of Engineering and Architecture, University of Trieste, Trieste, Italy; 2 Department of Cardiovascular Diseases, Laboratory for Cardiovascular Imaging and Dynamics, KU Leuven, Leuven, Belgium; 3 CMR Unit, Royal Brompton Hospital and Imperial College, London, UK; 4 Institute for Experimental and Translational Cardiovascular Imaging, DZHK Centre for Cardiovascular Imaging, Interdisciplinary Cardiovascular Imaging, Internal Medicine III and Institute for Diagnostic and Interventional Radiology, University Hospital Frankfurt, Main, Germany

**Keywords:** Cardiac mechanics, Feature tracking, Strain, Myocardial deformation, Cardiovascular magnetic resonance

## Abstract

Tissue tracking technology of routinely acquired cardiovascular magnetic resonance (CMR) cine acquisitions has increased the apparent ease and availability of non-invasive assessments of myocardial deformation in clinical research and practice. Its widespread availability thanks to the fact that this technology can in principle be applied on images that are part of every CMR or echocardiographic protocol. However, the two modalities are based on very different methods of image acquisition and reconstruction, each with their respective strengths and limitations. The image tracking methods applied are not necessarily directly comparable between the modalities, or with those based on dedicated CMR acquisitions for strain measurement such as tagging or displacement encoding. Here we describe the principles underlying the image tracking methods for CMR and echocardiography, and the translation of the resulting tracking estimates into parameters suited to describe myocardial mechanics. Technical limitations are presented with the objective of suggesting potential solutions that may allow informed and appropriate use in clinical applications.

## Background

Tissue tracking post processing for the calculation of myocardial deformation parameters can, in principle, be applied to the routinely acquired cross sectional images of cardiovascular magnetic resonance (CMR) or echocardiography studies. This makes the approach very appealing for clinical investigation and research, and increasing numbers of studies based on it are being published. Available software packages can be user friendly, readily delivering traces and measured outputs of deformation parameters. However, the methods used and their limitations may be insufficiently understood. Commercial distributors tend to not fully expose all methods used. This paper, written in collaboration between engineers and clinicians, sets out to explain the main principles and variants of methods applied. Exact implementation, however, may vary between packages, which may not be directly comparable with one another, or with other CMR based approaches to the quantification of myocardial deformation.

## Technology of tissue tracking

The technology of tissue tracking falls in a general category of image post-processing methods known as optical flow [1, 2]. The approach is broadly comparable to particle image velocimetry [3, 4] used in fluid dynamics. As with optical flow methods, the underlying principle is based on the recognition of patterns of features or irregularities in the image to be tracked and following them in the successive images of a sequence. This ‘feature tracking’ approach can be applied to routine cine CMR acquisitions [5] and is attracting the interest of many users in research and clinics. A comparable technique, commonly referred to as speckle tracking echocardiography (STE), has been widely applied in echocardiography where ventricular myocardium typically shows a speckled appearance [6, 7]. Optical flow methods need to be modified and optimized with respect to a particular field of application, with adjustments for image quality, temporal resolution, speed and magnitude of the expected displacements.

**Fig. 1:**
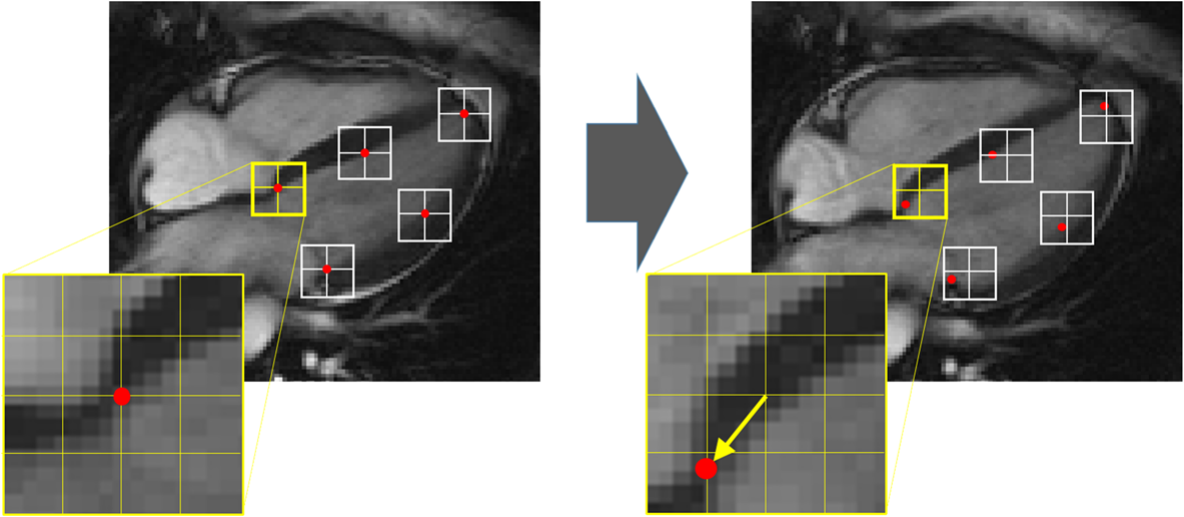
Basic principle of tissue tracking. Tracking the portions of tissue about a series of point (indicated in *red on the pictures*) is based on defining small square windows centered about such points on a first image (*left picture*) and searching the as-much-as-possible-similar grayscale pattern on the following image (*right picture*) in the vicinity of the original window. At the first step, shown here, the search windows have the same position in the pair of images and the feature that was at the center of the window of the first images is sought on the corresponding window on the second image (*red dot*) to provide an estimate of the local displacement. This procedure is usually repeated moving the second windows at the center of the new position and using a so-called coarse-to-fine approach, reducing progressively the window size. Large windows permit to recognize large gross displacements of the tissue, while the reduction of the window search area about the previous estimated targets allows to improve the accuracy and the locality of the estimation

In general, a tracking method begins by identifying a relatively small window on one image and searching for the most comparable image pattern in a window of the same size in the subsequent frame as shown in Fig. 1. The displacement found between the two patterns is taken as the local displacement of the tissue. A larger interrogation window will be needed if a pattern is displaced beyond the limits of a small search window, so window size may need to increase with the time interval between frames. However, use of a large window may reduce the accuracy of results because pattern similarities are averaged over a large area. While a small interrogation window may be unsuitable for detecting large displacements, it can improve the accuracy of detection of small ones. However, if a window is too small, it can become less feasible to recognize successive patterns [2]. In cardiology, speckles or features need to have the size of a few pixels to be recognized, and the dimensions of the window need to be at least 8 x 8 pixels.

The temporal resolution of the modality used is important. If too low, larger displacements will necessitate larger search areas, and the local patterns could become less comparable, an effect known as image de-correlation [8]. On the other hand, a very high temporal resolution must be accompanied by a high spatial resolution, otherwise frame to frame displacements may become smaller than the dimensions of pixels and harder to detect [3]. A complete tracking method may implement a hierarchical sequence of tracking steps. Initially, it detects the inward or outward motion of the cavity-tissue interface. It does this by taking the pattern of signal in the vicinity of each pre-selected point, typically along the endocardial border, located manually in one frame, and effectively shifting the pattern around the vicinity until the most closely matching pattern is found in the neighboring frames. Preferably, the boundary points are located in a frame near end systole as borders can usually be tracked more reliably when displaced apart. Conversely, if adjacent points and their search areas move together and overlap during contraction, the tracked border may depart from the visible boundary. In long-axis views, the relatively large motion of the atrio-ventricular junctions are commonly detected first by searching for the longitudinal displacements of relatively large image patterns in this region. The entire border is then adjusted in relation to this motion, proportionally from the base to the apex, which is assumed fixed at this stage. In the following stages, the algorithm refines the previously computed wide motion and detects local motion reducing progressively the search zone with smaller windows up to a minimal window size [9, 10].

The tracking technology is also amenable of integration with other techniques, like edge detection or registration methods, which can be used to adjust a tracked geometry toward predefined physiological shapes or according to elastic properties [11–14]. Implementation of such adjustments should be always described, because they aim to artificially improve estimation of tissue motion, for example by driving the solution toward an expected geometry which avoids unrealistic shapes or imposing expected elastic properties, at the same time they force a prescribed behavior may differ from the actual one and alter the strain pattern.

Tissue tracking was initially developed for two-dimensional (2D) images, but the technology can in principle be extended to track three-dimensional (3D) volumetric regions without conceptual differences. As a result, some 3D tissue tracking solutions are currently available although experience with them is still limited. When this extension is feasible, local 3D tissue features may be tracked simultaneously in all directions to derive all deformation parameters. This could theoretically reduce artifacts in deformation such as those that may result from through-plane of displacements of 3D structures.

### CMR feature tracking: strengths and limitations

Cine CMR is well suited for feature tracking (CMR-FT) by virtue of its relatively unrestricted access to large fields of view and its relatively high signal to noise and contrast to noise ratios. It provides the most accurate and reproducible assessments of global atrial and ventricular volumes and function available [15, 16]. Today’s standard steady state free precession (SSFP) techniques give good contrast between the myocardium [17] and the blood pool at a spatial resolution of about 1–2 mm in-plane and 6–10 mm through-plane acquired during breath holds of approximately 6–8 s. A limitation is the temporal resolution, which may not be able to resolve short-lived phases of cardiac motion. A standard CMR protocol covers the complete left and right ventricle with a stack of short axis images with additional long-axis views (usually 2-, 3- and 4-chamber views).

CMR does not appear to be able to distinguish features within the compact myocardium of the LV, presumably due to the relatively large dimensions of voxels and the relative homogeneity of water content and tissue properties within them. Tissue tracking in CMR is therefore most effective around endocardial borders, most of which are trabeculated. The epicardium can also be distinguished, although its clarity depends on the image properties of overlying structures. It should also be borne in mind that cine acquisitions are periodic. A cine loop representing an effectively averaged cardiac cycle is typically reconstructed from ECG gated data acquired over several cycles in a breath hold and delivering 25 to 50 reconstructed phases per heartbeat. The frame rate depends on heart rate and various acquisition parameters. Since MR acquisitions obtain data over several heart beats minor beat-to-beat differences are smoothed out which, in combination with suboptimal temporal resolution, will obscure rapid isovolumic phases and might lead to underestimation of displacement and strain values.

Clinical potential of CMR-FT has been recently described [18, 19]. However, it must be borne in mind that different methods have different strengths and limitations as they rely on different strategies for optimal tracking results: some use pure two-dimensional search windows of different sizes; others also include tracking by local M-mode representation along various directions that can be more efficient and accurate when motion is predominantly along one direction (like the longitudinal displacement of atrio-ventricular junctions). Some solutions focus on the endocardium first, which offers a more distinct interface with the cavity, and then on the epicardium. In any case, through-plane motion is a particular concern in this technique because CMR-FT is unable to track features that move out of plane in subsequent tracking frames.

3D tissue tracking can in principle be applied to CMR. However, the stack of short axis cines typically acquired for ventricular volume calculation is not well suited for this as the effective resolution is too low in the through-plane, long axis direction. Nevertheless, 3D acquisitions with comparable resolution in all three orthogonal directions are technically feasible. Although these have yet to be widely implemented, they can be achieved by using relatively long, navigated acquisitions and fast compressed sensing techniques.

### Speckle tracking echocardiography: strengths and limitations

Speckle tracking echocardiography (STE) was the first example of tissue tracking in cardiac imaging [20–22]. Images with a good spatial resolution (pixel size about 0.3 mm) combined with a frame rate above 60 Hz yield a theoretical accuracy in the estimation of strain within a few percent. In STE, the patterns to be tracked are provided by speckles, which are relatively stable acoustic markers that can be identified in the 2D images and originate from the back-scatter of ultrasound from myocardial tissue. Even though they are more evident at the endocardial border they are also present inside the myocardium which can be tracked directly.

Speckles are sites of positive interference with the ultrasound wave. They depend on the orientation of the scattering sites and the incident ultrasound field. The highly organized microstructure of compact myocardium consists of a syncytium of myofibers with a specific orientation, entangled with different types of collagen and organized in populations of locally oriented micro-laminar arrays known as sheets or sheetlets [23, 24]. We are not aware that the origin of speckles as a result of interference of scattering has been conclusively determined, but it is clear that this tissue organization, which includes locally oriented microstructural arrays, could potentially explain the differentiated back scattering of ultrasound by compact myocardium. The importance of the orientation of the scattering structures, such as fibers, sheets or collagen, with respect to the incident ultrasound beam, has been investigated [25]. Scattering structures are more echogenic when orientated perpendicular to the line of insonation than when they lie oblique or parallel to it.

During the cardiac cycle fibers, sheets and collagen will deform and the orientation of the scattering with respect to the incident ultrasound beam will change, leading to changes in interference patterns and therefore speckles. Because the deformation is relatively slow and coherent, the speckle patterns change relatively slowly and can be tracked over several cycles. But this is a reason for decorrelation in time and the need of a relatively high frame rate in STE.

Furthermore, echocardiography images have a lower signal-to-noise ratio than CMR. Some segments of the myocardium may not be adequately imaged and so require averaging of the measured values to fill these gaps. Indeed, a major limitation for the STE technique is that it is greatly dependent on the image quality, making it difficult in instances of poor echogenic windows, ultrasound dropouts and reverberations. In addition, because the quality is worse in the distal part of the ultrasound sector, more proximally located speckles enable better tracking. This leads to inconsistencies of accuracy and reproducibility, particularly relevant to segmental measurements and mitigated through the calculation of average, global strain [26].

Despite the suggested angle independency of STE, the best tracking quality can be achieved from speckles that move in the direction of the ultrasound beam [27, 28] where resolution is higher. Lateral resolution, perpendicular to the ultrasound beam, is lower and subjected to interpolation procedure across adjacent beams. Accordingly, apical views are more suitable for tracking speckles in the longitudinal direction than the radial direction, while parasternal short axis views give varied accuracy, according to segment, for radially or circumferentially directed strains. Averaging over the circumference may be preferable.

Echocardiography has a relatively high temporal resolution; but the selection of frame rates represents a tradeoff in the clinical settings where increasing the sector width for a larger field of view is accompanied by a decrease in frame rate if spatial resolution is maintained [26].

More recently, with advances in matrix-array ultrasound transducers and the development of 3D echocardiographic techniques, 3D speckle tracking (3D-STE) has become feasible and several solutions are currently available. However, 3D echocardiographic images have substantially (at least 3 to 4 times) lower spatial and temporal resolution than their 2D counterparts, and image quality may not be adequate. Currently, 3D-STE assessments remain controversial [27].

### Known pitfalls of the tissue tracking technology

One basic assumption underlying tissue tracking in 2D images is that apparent in-plane displacements of boundaries or deformations of gray-scale distributions correspond to actual displacements or deformations of tissue structure. This is not necessarily the case. For example, through-plane displacement of a tapering, helically structured or otherwise obliquely angulated forms could be misinterpreted as in-plane deformation or displacement in a 2D image. On the other hand, it is also true that the through-plane motion is small in most regions, and first experiences of 3D tracking do not report systematic differences in this sense [29, 30]. It is particularly in basal short axis or off-axis long axis planes that the user must remain aware of this potential effect and consequent potential misinterpretations.

Another assumption is that the myocardium is a coherent deformable tissue. Again this may not be true for all regions or across all spatial scales, for example in the trabeculated myocardial layer. Good spatial resolution is crucial to grasp the deformation of these small structures and problems arise with low-resolution images. In general, the use of relatively large interrogation windows in the tracking procedure helps to overcome this issue although it is not known whether effectively or only apparently.

Use of tissue tracking technology implies the underlying assumption that the motion detected on the image sequence represents the motion of the actual tissue and that blood motion is not visible in the image. This is usually true in echocardiography because ultrasound wavelength is too long to detect blood cells, unless a contrast agent (made of hyper-echogenic resonating bubbles) is injected. However, blood motion can be visible on cine CMR with potential to affect tracking near the endocardial border. Blood may moves in a direction opposed to that of tissue (for example from base to apex during diastole when tissue moves toward the base) and may give rise to unrealistic results. CMR-FT therefore needs to be used with caution where adjoining intra-cavity blood flow is clearly visible.

The most critical problem associated with any of the tissue tracking techniques appears to be suboptimal repeatability, even of the post processing, let alone between different studies and their operators [31–34]. The frame to frame tracking is based on the search for most probable pattern correspondence between regions in successive image frames. This delivers displacement values identified by being at the maximum of a correlation function. But these functions are generally smooth 2D surfaces (or 3D hypersurfaces in 3D tracking). Locating the maximum point along a smoothly bulging surface is not easy. It is comparable to asking 3 people to locate the top of a smooth hill: they may chose points of similar height but different location. Moreover, the actual position of such maxima can shift significantly when the surface is slightly modified by small variation of the search windows. Therefore, small differences of parameters or user choices during the tracking process can lead to different results. The presence of distinct features such as a sharp tissue-cavity interface or specifically delineated tags in a dedicated CMR acquisition increases the sharpness of the correlation functions and therefore reproducibility.

The problem of poor repeatability may be reduced by tracking procedures that incorporate multiple steps with additional calculations aimed at improving the accuracy of pattern location. Nevertheless, the user must still check the border motion visually to avoid evident errors, and possibly repeat the calculation to verify the consistency of the results found. End-user software tools may incorporate smoothing and validation procedures to reduce variability and minimise unrealistic results. On the other hand, these artificial steps could mask clinically significant abnormalities, either of localised regions in the case of spatial smoothing, or of rapid transients in the case of temporal smoothing.

Even under ideal conditions, the tissue tracking technology presents a technical limitation related to the pixel size. If a feature is displaced less than one pixel it may not appear to move. However, in practice, sub-pixel estimation can be achieved by testing larger, multi-pixel areas, although this may result in less accuracy and reproducibility. This limitation implies that an increase in the acquisition frame-rate improves the estimation of rapid displacements and large velocities but, at the same time reduces the accuracy in the evaluation of slow motion, if not accompanied by a corresponding increase in spatial resolution.

Finally, it must be kept in mind that the tissue tracking technology is based on ‘estimations’ of tissue displacement rather than on exact formulae; therefore the presence of small errors is unavoidable during the evaluation of relative motion. When these inaccuracies are additive over successive frames, errors can accrue in the displacements calculated. This issue can be partially corrected taking into account the beat-to-beat periodic motion of the cardiac tissue and correcting the drift by assuming that the tissue returns to the same position by the following end-diastole. Some solutions enforce the constraint of periodicity during application of the tracking algorithm rather than by correcting afterwards. The estimation errors are usually uncorrelated between the different regions and the different time instants. This implies that global results are more accurate than local ones because they describe an average process where uncorrelated errors partly cancel out. Similarly, instantaneous measures (e.g. velocity or strain-rate) are less accurate than time-integrated parameters like displacement or strain.

## Calculations of deformation parameters from tissue tracking

The attempted tracking of tissue motion from cine image frames is only a starting point for the quantification of cardiac deformation parameters. The tracked points are assumed to represent discrete tissue components that may move relative to given spatial coordinates. Relative to the most widely used ventricular coordinate system, they can move radially, which can usually be taken to be perpendicular to the local wall plane, and longitudinally or circumferentially, which mean along the wall as seen in either a long or short axis image plane, respectively. The tissue velocity vector can be computed as the frame-to-frame displacement divided by the time-interval. Bearing in mind that this is a time-derivative procedure (computed from the difference between displacement values at different instants), it is noisier than the displacement itself, reduces the accuracy, and requires a sufficiently high frame rate.

The knowledge of more than one region of tissue motion allows the calculation of tissue strain between them. Strain can be expressed either as a decimal fraction such as 0.15 or as a percentage such as 15 %. Expression as percentage can give rise to confusion and potential misinterpretation when it comes to reporting, as percentages, the relative differences of measurements between groups. Specific percentage differences could then have one of two meanings: absolute differences of strain measurements, or proportional differences between groups, so it is important to describe and interpret such results very carefully.

Strain records whether the length *L* of a piece of tissue gets smaller (shortening or thinning, known as negative strain) or larger (lengthening or thickening, known as positive strain) with respect to an initial end-diastolic length *L*_*0*_.

Based on this, strain can be defined as:

1 $$ St=\frac{L-{L}_0}{L_0}; $$

This is known as *Lagrangian strain* because it refers to an initial undeformed state [26]. In some applications a different definition, called *Eulerian strain*, has been used where the same difference in lengths is normalized with the final length *L*, instead of *L*_*0*_, in the denominator of (1); however use of this formula is not usual in cardiology.

Strain can be computed taking a tissue segment of length *L* along any specified direction. When this segment is taken along the longitudinal direction it gives the *longitudinal* strain, or *circumferential* strain when it is taken along the circumference, or it is the *radial* strain when the length is taken over the thickness. *Endocardial* longitudinal and circumferential strains are computed when the segment of tissue length *L* is taken from the endocardial border. Endocardial strain values are those more frequently used in clinical studies because they better represent the functional purpose of myocardial contraction, reducing the endocardial surface of the cavity to eject the stroke volume, and as such correlate best with volumetric measurements like ejection fraction [35]. Likewise epicardial strain, which it typically less than endocardial, can be evaluated along the epicardial border, although this is rarely used. It has been argued that strain is of interest as a measure of muscular contraction [36, 37], and it has been measured as the average value of strain computed over the myocardial thickness.

The description of displacements or strain must be expressed with respect to an initial, reference state, which is usually taken the end diastolic instant. In contrast, velocity and strain-rate should reflect an instantaneous activity irrespective of a starting point. From Lagrangian strain values the *Lagrangian strain-rate* can be computed as its time-derivative

2 $$ S{R}_L=\frac{dSt}{dt}=\frac{1}{L_0}\frac{dL}{dt}; $$

which means that, as for velocity, strain-rate is a differential quantity and its evaluation depends on sufficient temporal resolution. Its accuracy is lower than that of strain. However, looking at (2), it is apparent that the definition of Lagrangian strain-rate does depend on a reference length *L*_*0*_, a dependency that is disturbing when defining an instantaneous property. For this reason, Lagrangian strain-rate is not commonly used. Instead, a physically consistent definition for strain-rate is known as *natural strain*-*rate*

3 $$ SR=\frac{1}{L}\frac{dL}{dt}; $$

where the rate of shortening of a length *L* of tissue is measured relative to the actual length of tissue, independently from its previous deformation history.

The Lagrangian strain (1) and the natural strain-rate (3) are those more frequently used in cardiology, usually without the Lagrangian/natural suffix. In that case, the relationship between strain and strain-rate becomes

4 $$ SR=\frac{1}{St+1}\frac{dSt}{dt},St=exp\left\{{\displaystyle {\int}_{t_0}^tSRdt}\right\}-1. $$

This apparent complexity derives from the need of defining strain in the intuitive manner (1), while a natural definition of strain as the integral of the natural strain-rate, and commonly called *natural strain*

5 $$ S{t}_N={\displaystyle {\int}_{t_0}^tSR\ dt}= \log \frac{L}{L_0}, $$

would have ensured an improved mathematical consistency. But the definition (5) is less intuitive and cannot be immediately compared to visual measures, like thickening from M-mode, and is not practical for clinical cardiology.

### 3D deformation parameters and future representation

In principle, all deformation parameters can be computed from a single 3D acquisition by 3D tissue tracking. In 3D, three normal strain values (i.e., longitudinal, circumferential, and radial) can be computed at each point to describe the amount of shortening or stretch in the tissue. At the same time, the sliding between tissue layers at each point is described as shear (i.e., circumferential-longitudinal, longitudinal-radial and circumferential-radial shear values, respectively). An important shear component is the circumferential-longitudinal shear, which describes the torsional deformation of the LV. What is commonly referred as LV torsion is usually evaluated by the difference of rotation measured in the basal and apical short axis level (referred as LV twist), normalized with the distance between the two planes [38, 39]. This definition of LV torsion has the unit of degrees/cm, this is used in cardiology but it is not typical for other evaluations of shear that are generally defined as the ratio between two sides of a sheared parallelogram, giving a dimensionless measure representing the tangent of the shear angle.

In general, the strain and shear together describe the complete deformation and the individual values are arranged in a 3x3 table which is defined the *strain tensor*

6 $$ \mathbf{St} = \left[\begin{array}{ccc}\hfill S{t}_{long}\hfill & \hfill S{t}_{long- circ}\hfill & \hfill S{t}_{long- rad}\hfill \\ {}\hfill S{t}_{long- circ}\hfill & \hfill S{t}_{circ}\hfill & \hfill S{t}_{circ- rad}\hfill \\ {}\hfill S{t}_{long- rad}\hfill & \hfill S{t}_{circ- rad}\hfill & \hfill S{t}_{rad}\hfill \end{array}\right]; $$

where, for completeness, it must be remarked that the strain tensor (6) is symmetric because a non-symmetric contribution would correspond to local rigid rotations that do not contribute to deformation. Before moving ahead, it is useful to remark that different definitions of strain can be employed in the strain tensor (6). Lagrangian, Eulerian and natural strain were previously introduced in the context of two-dimensional imaging and can be immediately extended to 3D; other definitions are often employed to better relate the finite deformation to the effective tissue stress depending on tissue material properties [40]. This brief discussion, however, is limited to strain description without entering into tissue stresses and elasticity, and applies independently from the specific definitions adopted.

When considering 3D deformation in its entirety it is also possible to enforce the constraint of the conservation of mass or volume (neglecting the displacement of the intramyocardial blood volume). Contraction in longitudinal and circumferential directions is accompanied by radial thickening. In quantitative terms this means that the sum of the three strains values on the diagonal of (6) is equal to zero (this is true considering natural strain, otherwise the relationship is slightly more complicated); thus once two strain values are measured, the third can be recovered from first principles. In practice, tissue tracking in 3D is more easily performed at the endocardial level because it is aided by the echogenicity, or signal contrast, of the tissue-cavity interface, while the spatial resolution does not allow adequate resolution of variations across the thickness comprised only of a few pixels. In that case, the circumferential-longitudinal (torsional) shear is the only shear measured and the value of radial strain can theoretically be calculated based on the principle of conservation of mass.

It remains unclear how all of these 3D strain components can be interlinked and represented for convenient clinical assessment of myocardial deformation. One approach, initially developed through CMR technologies [41–43], and recently introduced in 3D echocardiography [44–46], entails identification of the *principal directions* of deformation. Principal directions are those orthogonal directions defined such that there is no shear across them. Therefore, the same 3D deformation, described in (6) with reference to the longitudinal circumferential and radial directions, can be equivalently described by only three principal strains along the principal directions without shear. CMR results showed that the greatest systolic principal strain is positive and directed predominantly in the radial direction, while the other two are predominantly in the wall plane and both negative, so delivering reduction of the LV cavity, with one contractile strain much larger than the other.

## Tissue tracking reliability and reproducibility

Tissue tracking software can give measurements of LV deformations rapidly and easily. However, given the limitations of the technique, users must be aware that it is not a perfect measurement tool.

First, 2D tissue tracking analysis should ideally be performed in defined planes through the LV where the tissue is well visualised, through-plane motion is minimal and image quality is high, with sufficient spatial and temporal resolution. Even then, results still have potential for variability. Therefore, multiple tracing, with visual checks for effective tracking, can help to reduce the risk of erroneous results. In particular, in long axis views, the tracking of the annulus must be checked visually as errors here can corrupt other parameters.

**Fig. 2:**
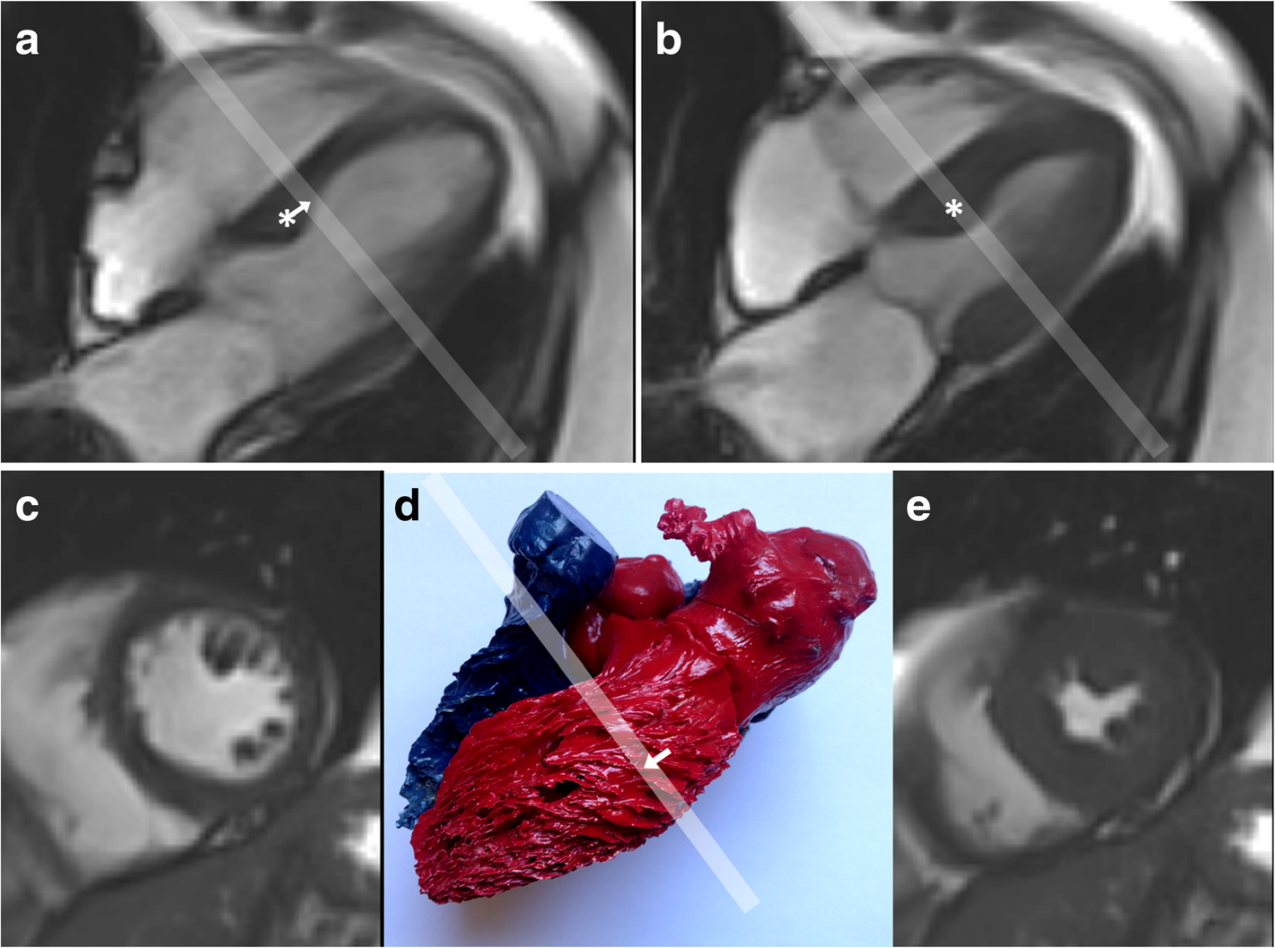
Possible effects on apparent strain of through-plane tissue displacements and trabecular appearances. Panel **a** This end-diastolic four chamber cine frame shows a basal septal bulge (*) whose long axis displacement causes it to move apically (*arrow*) to its end systolic position (**b**). Here it has moved into the short axis plane marked by the pale line. The septum in that short axis plane will therefore appear to have thickened more than it really did so that excess radial strain could be measured by tissue boundary tracking. Conversely, a more basal short axis slice might underestimate local radial strain due to tapering of the septum near the atrio-ventricular junction. Panel **c** Short axis images typically show trabeculations inside the LV free wall. Panel **d** this resin cast of human heart cavities shows the typical right handed helical alignments of free wall trabecular indentations. Systolic long axis displacement of these oblique trabecular structures (*arrow*) could give a false impression of trabecular displacement in the plane indicated by the *white bar*. A further issue is that trabeculations tend to thicken and move together in systole. This can exclude intervening blood, particularly in a hypertrophied ventricle with good function (panel **e**, from the same cine as **c**). If they merge to appear as part of the LV wall, it could result in over-estimation of radial and circumferential strain, if these were based on attempting to track the apparent endocardial boundary

Secondly, it must be understood that different variables and indicators of mechanical function present different reliability. As a general rule integral variables, like displacement and strain, are more reliable than differential ones like velocity and strain-rate, which require more cautious interpretation. Moreover, when the time interval between frames is large, velocities and strain-rate values cannot reproduce transient phenomena, such the spikes at iso-volumetric phases, that last no more than a few of such intervals. Among displacements, measurements of rotation can be less reliable because of the small displacements along the line of the wall relative to those of the inward, radially directed movement of the endocardial boundary. However, radial strain is usually less accurate than longitudinal and circumferential strain because the distance between endocardium and epicardium is small, there may be systolic elimination of visible blood spaces between trabeculae that exaggerates apparent shift of the endocardial boundary (Fig. 2), and there may be overlap of adjacent interrogation windows during calculation that makes it hard to resolve different displacements between adjacent regions. Table 1 gives an overview of the relative accuracies of different parameters of cardiac deformation.

**Table 1:** Parameters based on instantaneous and/or local results are less reliable than those based on a proper combination of a large number of results

More reliable (time-integral parameters)	Less reliable (instantaneous parameters)	Notes
Displacements	Velocities	Radial motion more accurate than tangential (e.g. rotation) because the tissue-cavity interfaces is a better feature that those found along the tissue.
Strain	Strain-rate	Values along the borders (longitudinal and circumferential strain) are more accurate than radial ones because the latter is a difference between close structures.
Properties of time-curve profiles (phase of harmonics, principal components)	Instantaneous values (peak, time to peak)	Instantaneous measures present unavoidable estimation errors which are smoothed-out when a larger number of measures is combined together.
More reliable (spatial-integral values)	Less reliable (local values)	
Global Strain	Segmental strain	Local measures present unavoidable estimation errors which are smoothed-out when a larger number of measures is combined together.
Measures built by numerous spatial values	Indicators built by values at single points	

Thirdly, with variable results, the application of smoothing can result in either regional or instantaneous values being less reliable. Currently, global measures give more reliable results. They include volumes, areas, global strain, average inward motion and possibly mean rotation. Regional differences may be better described in terms of mean dispersion (standard deviation) or other integral measures that are based on appropriate combinations of numerous values. A particular case is the visualisation and quantification of dyssynchronous motion [47]. Complete deformation curves through the course of the cycle can be taken into account when comparing the amplitude or timing of movements rather than confining measurements of point values such as a single peak or time to peak. When visually reviewing time curves, we naturally compare whole curves and can learn to interpret differences. For quantification, statistical comparisons of the curves, for example by manifold learning, enables abnormalities to be detected from the complete trace and could potentially increase robustness of the technique [48].

### Dedicated CMR strain acquisitions

CMR has a long-standing tradition of dedicated sequences and post-processing techniques for non-invasive measurement of myocardial deformation. CMR-based myocardial line tagging [49] was probably the first technique to assess regional myocardial deformation non-invasively. The original linear tagging approach was then augmented by tagging two orthogonally intersecting sets of lines mark rectangular grids in a 2D image by Spatial Modulation of Magnetization (SPAMM or C-SPAMM) techniques. These have been considered the gold standard for myocardial strain, although their limitations include the possible obscuring by tag lines of some endocardial borders, suboptimal temporal resolution and the additional, dedicated acquisition time. The tag lines represent the intersections with the image plane of sets of tag planes orientated orthogonal to it. For this reason, tagging is not subjected to the strain artifacts illustrated in Fig. 2 that result from through-plane displacements of tapering or obliquely orientated structures. The methods of quantifying deformation from tagged images are essentially similar to those of feature tracking. However, tags represent imposed features that are more regularly and clearly defined. They can therefore be tracked with more ease than natural features, enabling higher reproducibility with potential for regional differentiation of strain values [50]. The challenge of post-processing has been further reduced by the Harmonic Phase (HARP) algorithm, using the first harmonic peaks of the image’s k-space which has been carefully validated [51]. This potentially delivers information relating to all tissue areas, not just tag intersection points which allows some improvement of effective spatial resolution and the technique has been widely applied to assessments of circumferential strains.

An alternative way to assess cardiac motion with CMR is ‘Displacement Encoding with Stimulated Echos’ (DENSE) [52]. Tissue displacement is measured at the pixel level in three dimensions (2 in-plane and 1 trough plane). This technique has no visible tags, can be performed with high spatial resolution and provides black blood images with a good endocardial border definition. Post-processing is relatively easy and displacement is calculated on a pixel level. However, clinical research with this technique has been limited. Strain-encoded CMR (SENC) was introduced as an extension to HARP, in order to measure strain directed orthogonal to the image plane, for example longitudinal strain from a short axis acquisition [53], although its use has so far been limited.

### Speckle tracking and feature tracking experience and prospects

More experience is available with STE echocardiography to assess virtually any cardiac chamber. LV longitudinal, circumferential and radial strains and strain-rates in addition to LV rotational mechanics were calculated using both 2D-STE and 3D-STE. Global longitudinal strain (GLS) averaged from the apical views is the most robust and reproducible of the LV deformation parameters [54–57]. In CMR-FT the most consistent parameters was GCS, closely followed by GLS [32, 58]. In contrast, variations in GRS between studies were large. Segmental strain values show significantly greater variability, at levels which may be unacceptable for clinical use [18, 19]. Overall GCS compared best with measurements by CMR tagging with HARP post-processing [5]; GLS was compared to SENC [59] showing small bias and fair agreement for both RV and LV.

Several studies recently compared results from STE and CMR-FT with good agreement in values of GLS and GCS [60–63]. Circumferential strain may present improved reproducibility in CMR-FT than in STE presumably for the higher quality of CMR short axis images. The high echogenicity of the fibrous annulus enables more accurate results for longitudinal strain by STE. Agreement is lower for radial strain, which is probably less reliable for both modalities as previously discussed. However, comparability is still inconclusive in pathologic conditions and non-satisfactory for regional deformation [18].

Recent echocardiographic studies demonstrated that GLS is a sensitive marker of LV dysfunction [64], and therefore GLS is now considered a useful diagnostic and prognostic tool [65]. GLS is well estimated by STE because it depends principally from the motion of the mitral annulus, which is highly echogenic; moreover, the use of averaged strain overcame the limitations of non-visible segments in echography. In CMR long-axis views, the annulus shows a complex structure which deforms and rotates during the heartbeat possibly with a small through-plane component. Its tracking can sometime be elusive and care may be needed to verify the motion detected by CMR-FT to ensure that GLS value is accurate.

In contrast, literature is less conclusive about circumferential strain. The evaluation of GCS in echocardiography is less widely used as it requires additional short-axis recordings, STE literature reports considerable variability which might be caused by echoes from other structures lying in short-axis views. Further studies are needed to assess its clinical value. CMR short-axis cines, on the other hand, are typically of good quality with few problems related to image artifacts so preliminary studies suggest good feasibility of the tracking approach [19]. Longitudinal strain can also be relevant clinically as it is associated with the apical displacement of the mitral annulus. Circumferential strain tends to be more segmentally variable, reflecting local inward motion and thickening. Thus, CMR-FT could potentially distinguish the relative advantages of global or regional circumferential strain measurements. The clinical relevance of deformation imaging is discussed in [18] in relation to different pathological conditions.

An important source of variability in STE was attributed to differences between different vendor machine and software [66]. The existence of software-related variability induced scientific societies (American Society of Echocardiography, ASE, and European Association of Cardiovascular Imaging, EACVI) to call for a standardization of deformation imaging. A first objective has been that of providing a unique common definition of strain parameters agreed by most vendors [26] and explicitly of stating whether results apply to the endocardium or to the entire myocardial muscle. Another important objective has been the comparison of results obtained from the available software on the same groups of subjects in order to verify intra-vendor and inter-vendor reproducibility [34]. This effort is driving the product development of imaging companies to ensure a comparable technological ground where differences are attributable to the accuracy of the proprietary approach and the proposed parameters or visualization options. As an initial result, GLS has been recently included in the guidelines for the evaluation of LV diastolic function by echocardiography [67]. A similar issue is anticipated in CMR-FT where different vendors are offering independently developed solutions whose differences are reflected in the numeric values [19]. These are also likely to require studies of comparability, and hopefully progress towards the optimization and standardization protocols as well as the harmonization between STE and CRM-FT. A potential difficulty is associated with the reluctance of the distributors of commercial post-processing software to explain all details of the methods used, especially in the ongoing process of competitive development. This is understandable, and methods may anyway be complex and in the process of improvement. On the positive side, a commercial operation brings with it the benefits of dedicated, user-friendly product development, distribution and support. However, it may also be worth considering the potential advantages a non-commercial, open-source and research-funded approach to software development. However the field progresses, further refinement of software, more informed use of it, and carefully conducted studies are all likely to be needed for CMR-FT to become reliable and widely accepted for clinical application.

## Conclusions

The automated tracking and analysis of tissue motion in cine images is a relatively new and convenient tool which, if used with awareness of its limitations, can provide the clinician with quantitative information and visually informative temporal traces that together can become a valuable support to the diagnostic process. However, tissue tracking is not an exact measurement method and requires knowledge of its underlying methods for informed interpretation. With current technology, global parameters are those that show sufficient reproducibility to be potentially useful as clinical parameters, with measurements of longitudinal and circumferential deformations more reliable than radial ones. Identification and standardization of the most effective available solutions would be desirable to reduce inter-vendor variability and facilitate comparative research.
